# An omnidirectional visualization model of personalized gene regulatory networks

**DOI:** 10.1038/s41540-019-0116-1

**Published:** 2019-10-11

**Authors:** Chixiang Chen, Libo Jiang, Guifang Fu, Ming Wang, Yaqun Wang, Biyi Shen, Zhenqiu Liu, Zuoheng Wang, Wei Hou, Scott A. Berceli, Rongling Wu

**Affiliations:** 1 Center for Statistical Genetics, Departments of Public Health Sciences and Statistics, Pennsylvania State University, Hershey, PA 17033 USA; 2 Department of Public Health Sciences, Penn State College of Medicine, Hershey, PA 17033 USA; 3 Center for Computational Biology, College of Biological Sciences and Technology, Beijing Forestry University, Beijing, 100083 China; 4 Department of Mathematical Sciences, SUNY Binghamton University, Binghamton, NY 13902 USA; 5 Department of Biostatistics and Epidemiology, Rutgers School of Public Health, Piscataway, NJ 08854 USA; 6 Department of Biostatistics, Yale School of Public Health, New Heaven, CT 06520 USA; 7 Department of Family, Population & Preventive Medicine, Stony Brook School of Medicine, Stony Brook, NY 11794 USA; 8 Malcom Randall VA Medical Center, Gainesville, FL 32610 USA; 9 Department of Surgery, University of Florida, Box 100128, Gainesville, FL 32610 USA; 10 Department of Biomedical Engineering, University of Florida, Gainesville, FL 32610 USA

**Keywords:** Statistics, Programming language

## Abstract

Gene regulatory networks (GRNs) have been widely used as a fundamental tool to reveal the genomic mechanisms that underlie the individual’s response to environmental and developmental cues. Standard approaches infer GRNs as holistic graphs of gene co-expression, but such graphs cannot quantify how gene–gene interactions vary among individuals and how they alter structurally across spatiotemporal gradients. Here, we develop a general framework for inferring informative, dynamic, omnidirectional, and personalized networks (idopNetworks) from routine transcriptional experiments. This framework is constructed by a system of quasi-dynamic ordinary differential equations (qdODEs) derived from the combination of ecological and evolutionary theories. We reconstruct idopNetworks using genomic data from a surgical experiment and illustrate how network structure is associated with surgical response to infrainguinal vein bypass grafting and the outcome of grafting. idopNetworks may shed light on genotype–phenotype relationships and provide valuable information for personalized medicine.

## idopNetworks: How to predict biological snapshots

Networks are central to the functionality of biological systems. Understanding how the underlying components of a system are interconnected through biological principles to determine the system’s behavior and dynamics presents an interesting but challenging computational topic of research in modern medicine. A team led by Rongling Wu at Pennsylvania State University, in collaboration with researchers from multiple universities in US and abroad, developed an innovative tool to recover and infer dynamic gene regulatory networks from static omics data. This tool overcomes the significant limitation of existing approaches that can only infer a single structurally simple, aggregate network from a large number of samples. The new tool reconstructs informative, dynamic, omnidirectional, and personalized networks (idopNetworks), which can track and monitor how regulatory networks drive genotype–phenotype relationships and facilitate the use of network tools to precision medicine.

## Introduction

Gene regulatory networks (GRNs) have been thought to operate as the genomic mechanisms that guide the organism’s response to changes in their environment.^1,2^ One promising subject of research in modern biology and translational medicine is how to infer biologically realistic and statistically robust GRNs from increasingly available transcriptional data and link them to physiological, pathological, and clinical characteristics.^3–5^ A number of statistical approaches, such as Boolean networks,^6^ Bayesian networks,^7^ mutual information theory,^8,9^ and graphical models,^10^ have been developed for network inference, and these approaches visualize GRNs as probabilistic, undirected or unidirectional graphs, where each node represents a gene and edges depict relationships between genes. However, such graphs may not be sufficiently informative for charting the topological structure of a GRN because genes may regulate and also be regulated by other genes, with regulations in different signs and strengths and varying across time and space scales.^3,11^

As the time generalization of Bayesian networks, dynamic Bayesian networks (DBNs) can code cyclic, causally directed, and probabilistic interactions into networks through temporal interdependence, but their application is often impaired by the choice of granularity when time spaces vary.^12–14^ When gene networks are modeled by a system of time-derivative ordinary differential equations (ODEs), most of these issues can be addressed.^15–18^ The implementation of ODEs critically relies on high-density temporal expression data and parametric dynamic functions. The first condition is crucial for obtaining reasonable solutions of ODEs,^19^ but the collection of time-series data is extremely expensive and, more importantly, infeasible for many experiments, such as multi-tissue studies. The second condition is very difficult to justify, since gene expression is often stochastically fluctuated.^20,21^ To the end, despite its capacity to code bidirectional, signed, and weighted interactions into a fully informative network, the direct use of ODE networking can be very limited in practice.

Here, we develop a general framework that can recover any fully informative network without need of temporal data and parametric fitting. We analogize a gene network to being an ecological community composed of many interacting species, in which ecology theory and evolutionary game theory are at play.^22–24^ We integrate elements of these disciplines to derive a system of quasi-dynamic ODEs (qdODEs) that model and recover gene networks across samples. The qdODEs preserve the advantage of time-based ODEs and, in the meanwhile, possess and combine several additional valuable features. First, gene networks are regarded as temporal or spatial snapshots of biological processes.^25^ Our approach can monitor and predict how gene networks change dynamically in response to developmental and environmental cues. Second, it has been clear that genes involved in biological traits or processes are innumerable and, thus, the resulting regulatory networks should be high-dimensional or even ultrahigh-dimensional.^26^ Despite being highly challenging, our framework allows large but sparse networks to be reconstructed, providing a way for visualizing the omnidirectional mechanisms underlying biological complexities. Third, existing GRN inference approaches are developed to reconstruct an aggregate network from a large number of samples, such as individuals, tissues, or cell types. This may not be sufficient because networks as a biological process display great variability among samples and change dynamically along a spatiotemporal gradient. More recently, Kuijjer et al.^27^ have proposed a reverse-engineering approach for inferring and using sample-specific networks to reveal population heterogeneity, but it is unclear how their approach can reconstruct fully informative networks and, thereby, identify the genomic mechanisms for sample-dependent divergences. Our framework can extract individualized gene networks for each sample from any type of expression data and compare how network architecture varies among individuals, treatments, and cell/tissue types.

Taken together, our framework is equipped with a capacity to reconstruct informative, dynamic, omnidirectional, and personalized networks (idopNetworks) from standard genomic experiments. We test and validate the framework by analyzing genomic data of circulating monocytes from human infrainguinal vein bypass grafting, aimed at treating lower extremity arterial occlusive disease.^28^ The utility of the framework is also supported by a second vein graft experiment for rabbits.^29^ By reconstructing graft- and outcome-perturbed idopNetworks, we can potentially gain a mechanistic understanding of vein bypass graft success vs. failure.

## Theory construct

The theory for idopNetwork reconstruction is interdisciplinary, founded on the seamless integration of community ecology and evolutionary biology through mathematical and statistical reasoning. Each discipline contributes its distinct elements to a unified framework of statistical inference for gene networks.

### Niche theory of biodiversity

The concept of niche was first defined by Elton^30^ to describe the ecological components of a habitat related to a species’ tolerance and requirement. This concept has been generalized to explain biodiversity and species coexistence patterns in ecological communities.^31^ A gene network, residing in any biological entity, such as a cell, a tissue, or even an individual, can be viewed as an ecological community, in which the expression level of a constituent gene corresponds to the niche occupied by a species and niche differences form community diversity and stability. From a community ecology perspective, the total expression amount of all genes in the network reflects the carrying capacity of the entity to sustain these genes and supply them with essential resources or energy for their function,^32^ which are a mixture of many unknown factors. We define the total expression level of all genes on an entity as the expression index (EI) of this entity. This concept, similar to environmental index coined to describe the overall quality of site in terms of the accumulative growth of all plants,^33,34^ can describe the overall occupation of all genes to the entity. By aligning EI values in an ascending order, we can convert discrete entities to a series of continuous variables that help establish a system of ODEs.

In an ecological habitat, each organism needs to respond to, and in turn alters, the distribution of resources and competitors.^35^ For example, an organism would grow fast when resources are abundant, or when predators or parasites are scarce, and may limit access to resources by other organisms or provide a food source for predators. The types and numbers of environmental variables constituting the dimensions of a habitat vary from one species to another and the relative importance of particular environmental variables for a species may vary according to the geographic and biotic contexts.^36^ Thus, based on the niche theory of biodiversity, the relationship of the abundance of a particular species (part) with the total abundance of all species (whole) across graded habitats can potentially describe and predict the inherent compositional structure of an ecological community and its response to environmental change. This part-whole relationship, governed by the power scaling theory, has been observed to pervade biology; for example, the power equation can well explain how total leaf biomass scales allometrically with whole-plant biomass across different plants^37,38^ and how brain size of animals scales with whole-body mass across animals.^39,40^ We introduce this power scaling theory to model how the expression of individual genes (part) scales with the total expression of all genes across EIs through a system of ODEs.

### Evolutionary game theory of gene expression

In an ecological community where many species coexist, a species may adopt a cooperative or competitive decision to maximize its chance to access to resources.^41^ This phenomenon has also been well recognized at the cell level in both humans and rats,^42,43^ by which a cell determines a goal-directed decision-making based on its accrued knowledge of the environment. In an elegant study of stress impact, Friedman et al.^44^ identified the cells and networks that enable a rodent to choose an appropriate strategy of responsiveness after evaluating possible costs and benefits. Such rational choice reasoning may also guide how genes, located in the same cell, promote or inhibit each other in a complex network. In other words, gene–gene interactions can be modeled as a game in which one player may choose to compete or cooperate with its opponents in a quest to maximize its payoff. Classic game theory, pioneered by mathematical economists,^44^ suggests that such choices are not arbitrary, but rather include a rational judgement based on a gene’s own strategy and the strategies of other genes. However, it is extremely difficult or impossible to interrogate the rationality of genes, making a direct application of classic game theory to gene network inference infeasible. To address this issue, we introduce evolutionary game theory, a combination theory of game theory and evolutionary biology,^24^ which does not rely on the rationality assumption when it is used to study community dynamics and evolution. In an evolving population, any strategy used by an individual to maximize its payoff would be constrained by strategies of other individuals that also strive to maximize their own payoffs and, ultimately, this process through natural selection would optimize the structure and organization of the population, making it reach maximum (best response) payoff.^45^

### Mathematical integration of evolutionary game theory and niche biodiversity theory

**Fig. 1:**
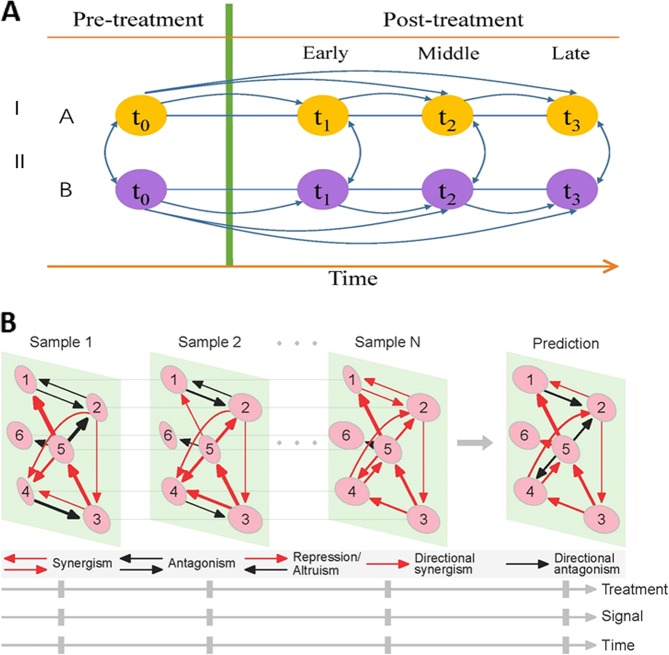
**a** Diagram of a standard genomic experiment under two levels of treatment, I and II. Transcriptomic profiles are monitored at key time points including one before treatment (*t*_0_) and those reflecting early (*t*_1_), middle (*t*_2_), and late stages (*t*_3_) of response after treatment. **b** Sample-specific visualization of idopNetworks reconstructed from the above genomic experiment, which describe how six genes co-regulate each other. Genes 1 and 2 are antagonistic in samples 1 and 2, but with different extents. These two genes are synergistic in sample *N* and altruistic in a predicted sample. The directional synergism of gene 5 to gene 1 is strong in samples 1 and *N*, but weak in sample 2. Because outgoing links are more than incoming links, gene 5 is a social gene in all samples, but the degree of its sociality is different across samples. Sample-specific networks can be compared between different treatment levels, signals (pre- vs. post-treatment), and times after the treatment

Suppose we initiate a standard genomic experiment (Fig. 1a) involving *S* treatments, each with *n*_*s*_ (*s* = 1, …, *S*) subjects, measured for *m* genes and *p* phenotypic traits at a series of time points (*t*_0_, *t*_1_, …, *t*_*T*_), where *t*_0_ denotes one pre-treatment time point and *t*_1_, …, *t*_*T*_ denote *T* post-treatment time points. We call a subject from a treatment measured at a time point a “sample.” Thus, we have a total of *N* = (*T* + 1)*n* samples, where $n = \mathop {\sum }\nolimits_{s = 1}^S n_s$ is the total number of subjects from all treatments. Let *M*_*ji*_ denote the expression level of gene *j* (*j* = 1, …, *m*) on sample *i* (*i* = 1, …, *N*). The EI of sample *i* is defined as $E_i = \mathop {\sum }\nolimits_{j = 1}^m M_{ji}$. We line up the *N* samples in the ascending order of EI, which allows us to construct a system of ODEs, expressed as

1 $$\frac{{dM_{ji}}}{{dE_i}} = g_j\left( {M_{ji}\left( {E_i} \right):{\mathrm{\Theta }}_j} \right) + \mathop {\sum }\limits_{j\prime = 1,j\prime \ne j}^m g_{j|j\prime }\left( {M_{j\prime i}\left( {E_i} \right):{\mathrm{\Theta }}_{j|j\prime }} \right),\,j = 1, \ldots ,m;i = 1, \ldots ,N$$

where the change rate of the expression of gene *j* per *E*_*i*_, *M*_*ji*_(*E*_*i*_), at a given sample *i*, is decomposed into the independent expression component, *g*_*j*_(·), specified by unknown parameters Θ_*j*_, and the dependent expression component, *g*_*j|j*′_(·), specified by unknown parameters Θ_*j|j*′_. The independent component of gene *j* occurs if this gene is assumed to be expressed in an isolated environment, and it is determined by this gene’s intrinsic property. The dependent component of gene *j* is the aggregated effect of all possible other genes *j*′ (*j*′ = 1, …, *m*; *j*′ ≠ *j*) on this gene. General speaking, the independent expression of a gene is determined by its endogenous encoding capacity, whereas its dependent expression is under the exogenous control. The structure of ODEs (1) is similar to the generalized Lotka-Volterra equations^46^ with the community matrix replaced by the functions $g_{j|j\prime }\left( \cdot \right)$ and the time derivative replaced by the EI derivative. Since they are not time based, such ODEs are called quasi-dynamic ODEs (qdODEs). It is straightforward to derive example equations of this type from the multi-gene replicator dynamics. Identifying these functions is a primary focus of research with a secondary effort being in interpretation and analysis of the resulting dynamical system.

### Inferring gene networks

In practice, the number of genes for network reconstruction is commonly very large (e.g., 10^3^–10^4^), thus if the expression of each gene involves the effects of all other genes, ODEs in Eq. (1) will quickly become intractable. Indeed, it is unlikely that each gene performs an interaction with every other gene in the network. By regressing the expression of each gene *j* on the expression of all other genes *j*′ (*j*′ = 1, …, *m*; *j*′ ≠ *j*), we formulate a multiple regression model across samples for variable selection. We implement adaptive LASSO to detect a small set of the most significant genes that affect a focal gene *j* (incoming links), but posing no constraint on the number of genes affected by the focal gene (outgoing links). This procedure enables the reconstruction of a high-dimensional but sparse and stable GRN under the convex optimization formulation (see the Methods). These networks (Fig. 1b) possess the following five features:

(i) *Bidirectional, signed, and weighted:* Let $G_j\left( \cdot \right)$ and $G_{j|j\prime }\left( \cdot \right)$ denote integrals of $g_j\left( \cdot \right)$ and $g_{j|j\prime }\left( \cdot \right)$ that constitute the system of qdODEs in Eq. (1), respectively. Note that, for a focal gene *j*, the number of its incoming links is *d*_*j*_ (<< *m*) after variable selection. The estimate of $G_{j|j\prime }\left( \cdot \right)$ can help judge in which way gene *j*′ affects gene *j*. If it is positive, negative, or zero, then this suggests that gene *j*′ promotes, inhibits, or is neutral to, gene *j*, respectively. The value of the estimate can quantify the strength of promotion or inhibition. By comparing $G_{j|j\prime }\left( \cdot \right)$ and $G_{j\prime |j}\left( \cdot \right)$, we can determine whether these two genes reciprocally trigger impacts on each other. Further, we reconstruct a bidirectional, signed, and weighted graph as the gene network of the sample by considering all possible gene pairs detected from variable selection. The estimate of $G_j\left( \cdot \right)$ represents how much amount of expression a given gene *j* may intrinsically release, and its value is proportional to the size of a node in the graph.

(ii) *Dynamic:* The amount of dependent expression $G_{j|j\prime }\left( \cdot \right)$ is a function of *E*_*i*_, suggesting that the dependent amount of gene *j* affected by gene *j*′ can be estimated at any given EI. Thus, we can reconstruct a series of “dynamic” networks across samples. These networks allow geneticists to test how GRNs alter structurally and functionally in response to environmental and developmental cues. These tests can be made locally, i.e., testing how networks differ between two time points of interest under the same treatment or between different treatments at the same time point.

(iii) *Omnidirectional but sparse:* If the number of genes for network reconstruction is large, we should build a high-dimensional set of ODEs that can specify the whole picture of gene interactions in the network. The implementation of variable selection can detect the most significant links to construct a sparse network but still allows all possible realistically existing links to be encapsulated as a whole that underlie the behavior of gene networks. This dimension reduction procedure will become even more valuable since more and more studies attempt to reconstruct regulatory networks from genomic, proteomic, and metabolomics data. A more fine-grained network inferred from these omics data at different levels or through different pathways can reveal previously hidden contributions of gene interactions to cellular processes.

(iv) *Personalized:* The most noticeable advantage of our approach is the ability to pack steady-state expression data into fully informative networks that can currently be inferred only from high-density temporal data. As a function of *E*_*i*_, the independent and dependent expression values of genes can be calculated for any sample from *G*_*j*_(·) and *G*_*j|j*′_(·), respectively. These values enable the inference of sample-specific networks from which to compare how networks differ among entities (e.g., subjects, tissue types, or cell types), treatment levels, and times (Fig. 1b). The main merit of a mathematical model is its ability to make a prediction for the future. The qdODEs allow the independent and dependent expression levels of genes to be calculated as long as EI is provided. Thus, for those samples that are not included in our network reconstruction, we can interpolate or extrapolate gene networks based on their EIs. Individualized networks are likely to be associated with clinical and disease phenotypes and, therefore, can be potentially useful for predicting health risk.

(v) *Biologically meaningful and socially interpretable*: Because of bidirectional and signed features, the network can discern distinct patterns of gene interactions (Fig. 1b). If two genes facilitate each other by producing factors that promote both parties, then synergism occurs. In contrast, an antagonism occurs if two genes inhibit each other. Directional synergism results if one gene promotes its partner but the latter does not affect the former (neutral), while directional antagonism occurs if one gene inhibits the other and the other is neutral. If one gene inhibits the other but the latter promotes the former, then the former exerts synergistic repression to the latter. Conversely, one gene promotes the other but the latter inhibits the former, then the former offers antagonistic altruism to the latter. A lack of any interaction, then, is when two genes coexist and are neutral to each other. These interaction patterns contain the underlying mass, energetic, or signal basis of gene interactions and, therefore, they are more biologically meaningful than the traditional description of gene–gene interaction based on statistical tests. A gene may actively manipulate other genes (by promoting or inhibiting the latter) but, meanwhile, may also be passively manipulated by other genes. In networks reconstructed from our approach, one can identify the numbers of such active links and passive links for each gene. If a gene has more active links than passive links, it is regarded as a social gene. If a gene’s active links are more than the average of all genes (i.e., connectivity), then this gene is a core gene that is believed to play a pivotal role in maintaining gene networks. If a gene has less links, including active and passive, than the average, it is a solitary gene.

## Results

### Human vein bypass grafting

Rehfuss et al.^28^ reported a genomic study of infrainguinal vein bypass grafting involving 48 patients, among whom 35 succeeded and 13 failed. To investigate the genomic mechanisms underlying graft outcome, transcriptomes of circulating monocytes from patients of success and failure were monitored at pre-operation and at days 1, 7, and 28 post-operation. We selected a subset of genes measured (1870) that change significantly as a function of time per ANOVA (*P* < 0.05) for idopNetwork reconstruction. Four time points of gene monitoring for 48 patients form 4 × 48 = 192 samples.

**Fig. 2:**
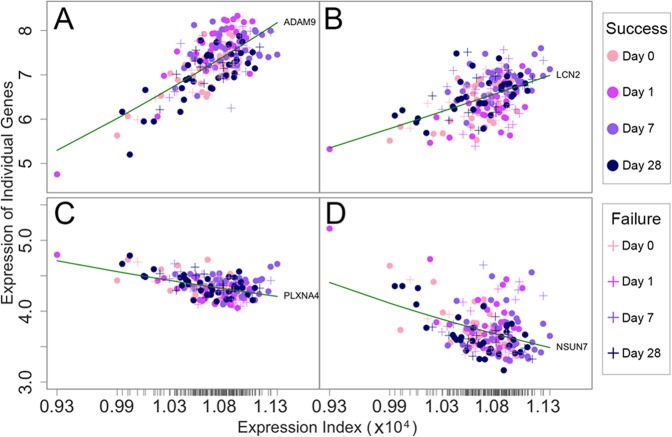
The fitness of a power equation as a function of expression index (EI) (green line) to the observed expression levels of four genes, ADAM9 (**a**), LCN2 (**b**), PLXNA4 (**c**), and NSUN7 (**d**), randomly chosen from the genomic study of human infrainguinal vein bypass grafting, across samples. Samples involve 48 patients, i.e., 35 successes (circle) and 13 failures (plus), multiplied by four time points (including day 0 pre-operation and days 1, 7, and 28 post-operation). Ticks on the *x*-axis represent the positions of each sample in terms of its EI

By plotting the expression of individual genes against EI across these samples, we found that each gene’s EI-varying expression is broadly in agreement with part-whole relationship theory. Figure 2 illustrates the goodness-of-fit of gene expression to the power equation by using four randomly chosen genes as examples. The expression of ADAM9 and LCN2 increases with EI, but the former displays a greater slope of increase (Fig. 2a) than does the latter (Fig. 2b). In contrast, the expression of PLXNA4 (Fig. 2c) and NSUN7 (Fig. 2d) decreases with EI, but with different slopes. The existence of allometric power equation suggests that gene expression can be described as a continuous function of EI, which is the basis for formulating qdODEs (1). This also facilitates the use of Kim et al.’s functional clustering^47^ to categorize all genes considered into different modules, each with a distinct EI-varying pattern. We found 145 such modules based on AIC. Table S1 lists the names of genes that are assigned into each modules. We reconstructed 145-node idopNetworks at the module level.

**Fig. 3:**
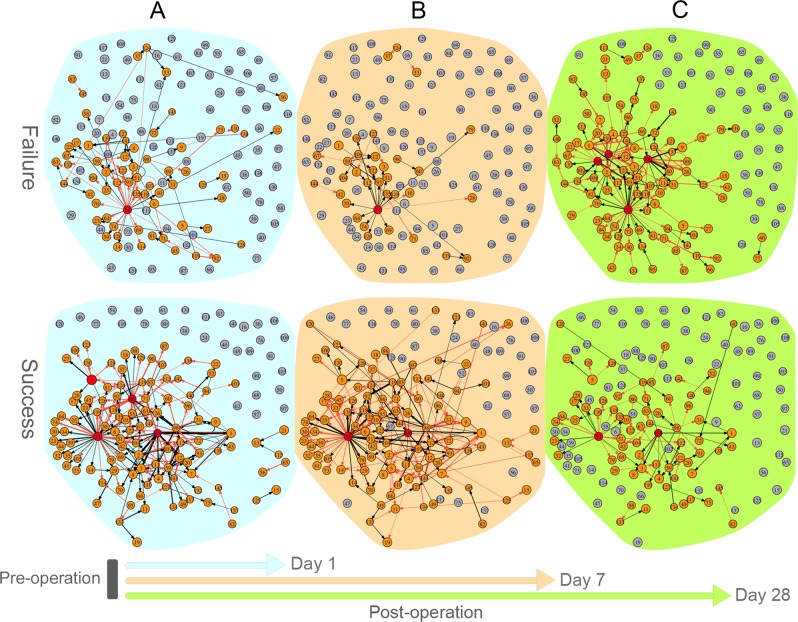
Graft-perturbed networks that code how different gene modules are co-expressed for a failed patient (upper panel) and a successful patient (lower panel) in response to physiological changes from pre-operation to day 1 (**a**), 7 (**b**), and 28 (**c**) post-operation. Numbers in small circles (each denoted as a node of the graph) represent module IDs. Red and blue arrows denote the direction by a gene promotes and inhibits other genes, respectively, and the thickness of an arrowed line is proportional to the strength of promotion or inhibition. A proportion of modules are unlinked, suggesting that they are neutral to each other and other linked genes. Dark red circles denote hub modules with higher connectivity than the average number of links among all modules

One major advantage of our model lies in its capacity to reconstruct personalized networks. In other words, our model can infer a specific network for each patient and monitor how this personalized network changes in response to environmental and developmental signals. To show this capacity, we randomly chose one successful patient (#125) and one failed patient (#205) and compare how they respond to grafting through network alterations. GRNs that specify the alterations of gene co-expression across environmental change are called environment-perturbed GRNs. Figure 3 illustrates graft-perturbed idopNetworks at the module level from pre-operation to days 1 (A), 7 (B), and 28 (C) post-operation, respectively, for parent #205 (upper panel) and #125 (lower panel). The two patients display some commonalities and differences in terms of their network structure and sparsity. For example, module 53 is a hub that actively regulate many other modules in both success and failure graft-perturbed GRNs. This module only contains an antisense lncRNA gene, C5orf26/EPB41L4A-AS1, located in the 5q22.2 region of the genome.^48^ This gene plays a role in the development, activation, and effector functions of immune cells.^49,50^ However, the two networks are remarkably different in many aspects. First, the success network contains more links than the failure network at the early and middle stage of recovery after grafting, but this difference disappears at the late stage of recovery, suggesting that the successful patient can more quickly establish a stable network than the failed patient. Second, the success network from pre-operation to day 1 post-operation is framed by multiple hubs (including not only 53 but also 5, 86, and 109), each displaying strong links with many other modules, but the failure network is only dominated by hub 53 with relatively weak links to other modules. Third, graft-perturbed networks alter more dramatically in topological structure across time for the failed patient than the successful patient.

**Fig. 4:**
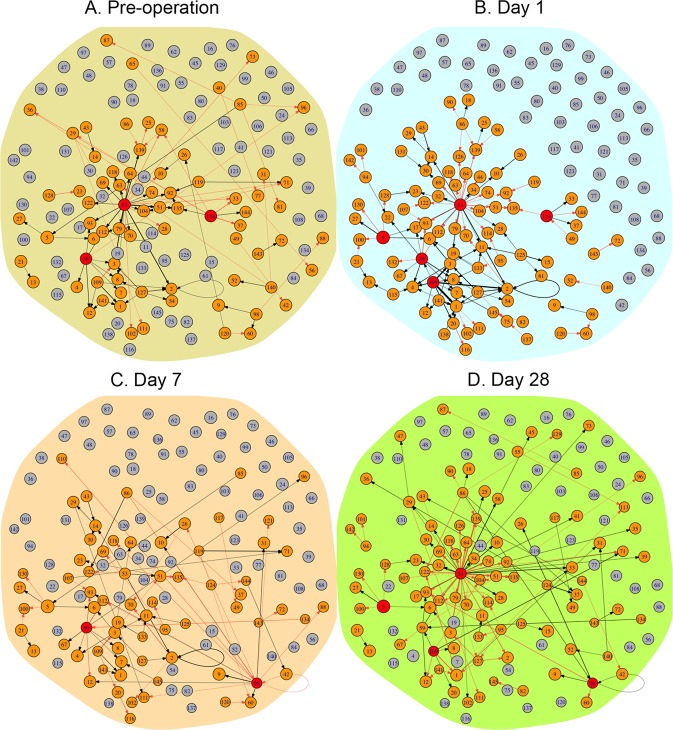
Outcome-perturbed networks that code how different gene modules are co-expressed in response to a successful patient vs. a failed patient prior to operation (**a**) and day 1 (**b**), 7 (**c**), and 28 (**d**) post-operation. Numbers in small circles (each denoted as a node of the graph) represent module IDs. Red and blue arrows denote the direction by a gene promotes and inhibits other genes, respectively, and the thickness of an arrowed line is proportional to the strength of promotion or inhibition. A proportion of modules are unlinked, suggesting that they are neutral to each other and other linked genes. Dark red circles denote hub modules with higher connectivity than the average number of links among all modules

Our model can also characterize whether network topologies can interpret overall differences between succeeded and failed patients. By averaging the networks of all successful patients and the networks of all failed patients, we reconstructed outcome-perturbed networks at different stages of operation (Fig. 4). We argue that if networks are not associated with graft outcomes, outcome-perturbed networks should be similar structurally between pre- and post-operation. The outcome-perturbed network prior to operation is dominated primarily by hub module 53, followed by module 124 (Fig. 4a), but the outcome-perturbed network at day 1 post-operation involves hubs 53, 124, 109, 59, and 5 (Fig. 4b). Module 53 drives the prior network purely through inhibiting other modules, whereas much of its role in the post network is played by promotion. Outcome-perturbed networks at days 7 (Fig. 4c) and 28 post-operation (Fig. 4d) differ not only from that prior to operation in terms of the number and type of hub modules, but also are sharply contrast to those at day 1 post-operation. Taken together, the genomic differences driving outcomes can be interrogated by the topology of graft- and outcome-perturbed idopNetworks reconstructed by our model.

**Fig. 5:**
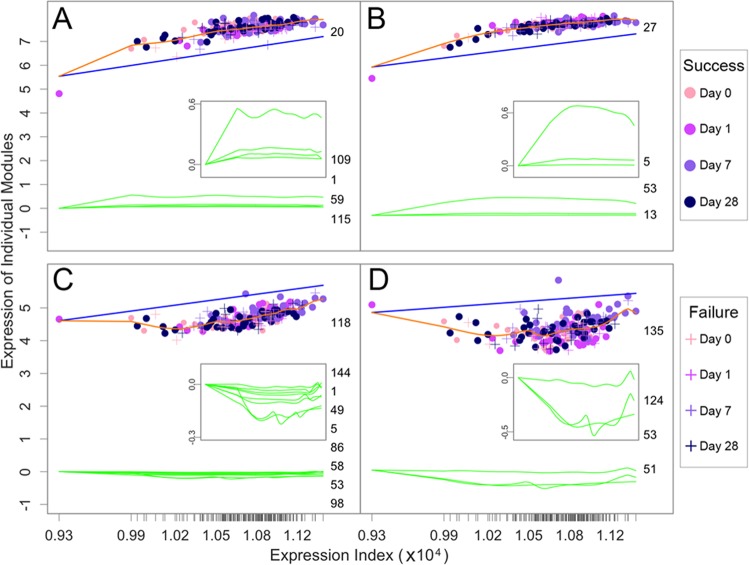
Overall fitted curves of gene expression (orange line) from modules 20 (**a**), 27 (**b**), 118 (**c**), and 135 (**d**) by a system of qdODEs as a function of expression index (EI) in the human vein grafting study. Each dot denotes a sample representing a patient with outcome success (circle) or failure (plus), measured at a time point (day 0 pre-operation and days 1, 7, and 28 post-operation). The overall expression curve of each module is decomposed into its endogenous expression curve (blue line) and exogenous expression curves (green lines) exerted by a set of other modules (listed by their IDs). Exogenous expression curves are better displayed by a small plot within each large plot. Value 0 at *y*-axis is a cutoff point that describes how a focal module is regulated by other modules: Greater than 0 for promotion, less than 0 for inhibition, and zero for neutrality. Ticks on the *x*-axis represent the positions of each sample in terms of its EI

How much a gene is expressed across dynamic networks is determined by its endogenous encoding force (independent expression) and the exogenous influence by other genes (dependent expression). Our model can dissect the overall expression level of each gene into its independent and dependent expression components. The sign and size of the dependent components can explain how each gene is regulated by other genes in the networks. Four representative modules 20, 27, 118, and 135 exhibit distinct expression patterns across samples, whose underpinnings can be illustrated by drawing the independent and dependent expression curves (Fig. 5). The independent expression of each module increases exponentially with EI, but the slopes of increase vary depending on module type. Modules 20 and 27 are each promoted by other modules, 109, 1, 59, and 115 for the former (Fig. 5a) and 5, 53, and 13 for the latter (Fig. 5b), both listed in the order of promotion degree. These modules produce accumulative positive dependent effects on the expression of modules 20 and 27, leading the observed expression level of these two focal modules to be higher than their independent expression level across EI gradients. By contrast, the independent expression level of modules 118 and 135 is downshifted by a set of eight modules for the former (Fig. 5c) and a set of four modules for the latter (Fig. 5d). These two sets of modules inhibit the expression of modules 118 and 135, respectively, producing accumulative negative dependent effects on the focal modules.

### Rabbit vein bypass graft

We analyzed a second data set of gene expression to validate the usefulness of our approach. The data of microarray genes was collected from a rabbit bilateral vein graft construct.^29^ New Zealand white rabbits (weighing 3.0–3.5 kg) of high genetic similarity were treated by bilateral jugular vein interposition grafting and unilateral distal carotid artery branch ligation to create two 6-fold different blood flows. Thousands of genes were monitored on vein grafts, harvested at 2 h, 1, 3, 7, 14, 30, 90, and 180 days after implantation, under both conditions, high flow and low flow. Each outcome involves three to six rabbits at each time point, which totalize 73 samples. We chose a set of differentially expressed genes (1395) for idopNetwork reconstruction. We calculated the EI of each sample with these genes and plotted the expression of individual genes against EI. EI-varying expression profiles, fitted by a power function (Fig. S1), were clustered into 50 modules (Fig. S1).

We reconstructed module-based idopNetworks of gene co-expression altered from time 2 h to 1 (A), 30 (B), and 180 days (C) after implantation under high and low flows (Fig. S2). These networks change strikingly in the structure and connectivity across times under both flow conditions. Also, at the same time, idopNetworks differ between high and low flows. Flow-perturbed networks are structurally simple at time 2 h, but show increasing complexities with time (Fig. S3), suggesting that high and low flows need a time to display their differences. Figure S4 illustrates how the expression of four modules is determined by their endogenous capacity and the exogenous influence of other modules. The overall expression of modules 3 (A), 45 (B), and 38 (D) was observed to be higher than their independent expression because of positive influences exerted by other modules, but module 20 (C) is negatively affected by other modules, making its overall expression lower than independent expression. Taken together, results from the rabbit grafting study support the usefulness of our network inference approach.

### Computer simulation

We performed computer simulation studies to empirically evaluate the accuracy of the proposed qdODEs approach. We simulated the expression data of *m* genes, ${\mathbf{y}}_j = \left( {y_j\left( {E_1} \right), \ldots ,y_j\left( {E_N} \right)} \right)\,\left( {j = 1, \ldots ,m} \right)$, across *N* samples, with *y*_*j*_(*E*_*i*_) varying with $E_i\,\left( {i = 1, \ldots ,N} \right)$. We let *N* change from 50 to 100 to 200. The EI-varying expression change of gene *j* was generated by a multivariate normal distribution with zero mean vector and covariance matrix following the AR(1) model. Each gene was designed to interact with a specific set of genes following a system of EI-varying qdODEs. To assess the robustness of the approach, we also changed the variance and correlation coefficients to control four different levels of variation for each fixed sample size, which generate a total of 12 scenarios.

The performance of the proposed approach was assessed by three criteria as follows:

*Sensitivity* quantifying the percentage of patients with the disease test positive, which is defined as

$${\mathrm{Sensitivity}} = \frac{{{\mathrm{TP}}}}{{{\mathrm{TP + FN}}}},$$

where TP is true positive and FN is false negative, and *specificity* measuring the percentage of healthy people test positive as

$${\mathrm{Specificity}} = \frac{{{\mathrm{TN}}}}{{{\mathrm{TN + FP}}}}.$$

where FP is false positive and TN is true negative.

Alternatively, we also computed the *positive likelihood ratio (PLR)* to give a comprehensive assessment

$${\mathrm{Positive}}\,{\mathrm{likelihood}}\,{\mathrm{ratio}} = \frac{{{\mathrm{TP/(TP + FN)}}}}{{{\mathrm{FP/(TN + FP)}}}}.$$

In general, a sensitive test picks up most of the patients with the disease but may also pick up healthy people without disease. A specific test will not pick up healthy people but may also miss a lot of true patients with disease. The trade-off between the sensitivity and specificity was balanced by maximizing the area under the receiver operating characteristic (ROC) curve (i.e., AUC). As shown in Table 1, both the sensitivity and specificity of the proposed approach are high, which indicate its accuracy and potential in clinical genomic studies. In addition, high positive likelihood ratios also show that the approach is very discriminatory. For example, a value of 21.706 means that a patient with the disease has a 21 times higher chance to be tested as positive compared with a healthy people.

**Table 1:** Statistical properties of idopNetwork reconstruction under different simulation scenarios

Sample size *N*	Variance/correlation coefficient	Sensitivity	Specificity	PLR
50	0.1/0.3	0.738	0.966	21.706
50	0.1/0.0	0.741	0.966	21.794
50	1.0/0.3	0.501	0.966	14.735
50	1.0/0.0	0.503	0.966	14.794
100	0.1/0.3	0.797	0.967	24.151
100	0.1/0.0	0.809	0.967	24.515
100	1.0/0.3	0.604	0.964	16.778
100	1.0/0.0	0.598	0.964	16.611
200	0.1/0.3	0.871	0.968	27.219
200	0.1/0.0	0.863	0.969	27.839
200	1.0/0.3	0.690	0.963	18.648
200	1.0/0.0	0.693	0.963	18.729

Something worth to mention is that the approach is quite robust with stable results among 12 combinations of different sample sizes and variations. When sample size is larger (*n* = 200) the results are the best, but neither specificity nor sensitivity reduces much if the sample size is only ¼ of it (*n* = 50).

## Discussion

A fully informative network is defined as one that encapsulates bidirectional, signed, and weighted interactions into a graph. Such networks can provide a detailed mechanistic understanding of how genes interact and work together to determine complex phenotypes. To reconstruct fully informative networks, existing approaches need the collection of high-density temporal data and the parametric fitting of the observed data, both of which are hardly met in many genomic studies. In this study, we develop a novel approach that can infer fully informative networks, not relying on these two conditions. The key component of our approach is a system of qdODEs that are derived by integrating elements of ecology theory and evolutionary game theory. The optimization solution of the qdODEs, through the implementation of variable selection, enables the inference and recovery of informative (encapsulating bidirectional, signed, and weighed links), dynamic (tracing network alterations across spatiotemporal gradients), omnidirectional (capturing all possible links but maintaining the sparsity of networks), and personalized (individualizing networks for each subject) networks (idopNetworks).

Vein bypass grafting is an essential treatment for lower extremity arterial occlusive disease, but only with 30–50% success rate.^28^ The biological mechanisms underlying the outcome of grafts include cue-induced differentiation of gene expression. We used our approach to reconstruct graft- and outcome-perturbed idopNetworks from 1870 differentially expressed genes, providing a new avenue to find key genes and key interactions that cause success vs. failure. We found that, as an antisense lncRNA gene, located in the 5q22.2 region of the genome, C5orf26/EPB41L4A-AS1^48^ plays a leadership role in regulating other genes within networks. How many genes it regulates, how differently it regulate these genes, and how its regulation responds to grafting and recovery may be potentially important for patients to cure. Based on previous functional studies,^49^ we postulate that the role of C5orf26/EPB41L4A-AS1 in mediating and activating the gene networks toward cure may be executed through its effects on the development, activation, and effector functions of immune cells. We found more links in the networks of successes than those of failures at the early and middle stage of recovery after grafting. Previous ecological studies show that the number of links, which is usually defined as the complexity of a network,^51^ is positively correlated with the stability of the network.^52–54^ This thus suggest that the successful patient can more quickly establish a stable network than the failed patient.

The past two decades have witnessed countless transcriptional experiments initiated to explore the genomic mechanisms underlying high-order phenotypes for a wide range of organisms. These experiments were designed to monitor gene expression profiles of biological entities under contrast conditions and/or across developmental times. By various comparative analysis and tests, genes expressed differentially under different conditions or over times are identified as biomarkers of phenotypic variation. Cluster analysis was also used to detect distinct patterns of gene expression, facilitating the interpretation of the genomic control over phenotypic or developmental plasticity. However, gene regulatory networks, despite their role in linking genotype to phenotype,^1,2^ have not been reported from a majority of genomic experiments. Now, our approach can recover and reconstruct idopNetworks using these publicly available data, from which to generate new discoveries traditional approaches fail to identify.

Our qdODE approach has great power to explore various omics data, generate mechanistic hypotheses, and guide further experiments, model development, and analyses. By validating or invalidating various hypotheses experimentally, new scientific discoveries can be made, new insights gained, and new network models revised. Our approach can be refined to accommodate the data features of single cell analysis,^55^ which facilitates idopNetworks to explore an in-depth mechanisms that drive remote biochemical, developmental, and physiological transitions from genotype to phenotype.

## Methods

The methods were performed in compliance with relevant guidelines and regulations, and approved by University of Florida. We obtained a written consent from the participants.

In what follows, we describe a statistical procedure for solving a system of qdODEs in Eq. (1). By obtaining the maximum likelihood estimates of independent and dependent expression amounts of each gene, idopNetworks can be reconstructed.

### Variable selection for interacting genes

Let **y**_*j*_ *=* (*y*_*j*_(*E*_1_), …, *y*_*j*_(*E*_*N*_)) denote a vector of observed expression values for gene *j* (*j* *=* 1, …, *m*) over all samples. The observed expression of gene *j* at sample *i* is expressed as

2A $$\begin{array}{l}y_j\left( {E_i} \right) = M_j\left( {E_i} \right) + e_j\left( {E_i} \right)\\ = G_j\left( {M_{ji}\left( {E_i} \right):{\mathrm{\Theta }}_j} \right) + \mathop {\sum }\limits_{j\prime = 1,j\prime \ne j}^m G_{j|j\prime }\left( {M_{j\prime i}\left( {E_i} \right):{\mathrm{\Theta }}_{j|j\prime }} \right) + e_j\left( {E_i} \right)\end{array}$$

2B $$= a_j\left( {E_i} \right) + X_j^T{\mathbf{b}}_j\left( {E_i} \right) + e_j\left( {E_i} \right),$$

where the overall expression level of focal gene *j*, *M*_*j*_(*E*_*i*_), includes its independent expression component, *a*_*j*_(*E*_*i*_) = *G*_*j*_(·) and dependent expression component accumulatively determined by all other genes, $X_j^T{\mathbf{b}}_j\left( {E_i} \right) = \mathop {\sum }\nolimits_{j\prime = 1,j\prime \ne j}^m G_{j|j\prime }\left( \cdot \right)$; the derivatives of *G*_*j*_(·) and *G*_*j|j*′_(·) are *g*_*j*_(·) and *g*_*j|j*′_(·) of ODEs in Eq. (1), respectively; and *e*_*j*_(*E*_*i*_) is the measurement error at sample *i*, assumed to be iid with mean zero and variance $\sigma _i^2$. Note that $X_j^T$ is the vector containing *m* – 1 unities and **b**_*j*_(*E*_*i*_) = (*b*_*j|*1_(*E*_*i*_), …, *b*_*j|m*_(*E*_*i*_)) is a vector of the dependent expression of gene *j* determined by all genes, except for gene *j*.

Many nonparametric functions, such as B-spline, regression B-spline, penalized B-spline, local polynomials, or Legendre orthogonal polynomials (LOP), can be used to model independent expression curves, *a*_*j*_(*E*_*i*_), and dependent expression curves, **b**_*j*_(*E*_*i*_). Chen et al.^12^ have proved statistical properties of B-spline variable selection for solving ODEs. Here, we implement B-spline to fit *a*_*j*_(*E*_*i*_) and **b**_*j*_(*E*_*i*_) in Eq. (2B), allowing orders of nonparametric functions to be gene-dependent and also differ between independent and dependent expression curves. For any gene *j* as a response, there are (*m* – 1) predictors, each of which contributes to the dependent expression of this focal gene through unknown nonparametric dependent parameters **β**_*j*_ **=** (**β**_*j*|1_,…,**β**_*j*|(*j*–1)_,**β**_*i*|(*j* + 1)_,…,**β**_*j*|*m*_). Thus, we have *m* – 1 groups of dependent parameters that reflects the regulation of other genes for the focal gene. We implemented group LASSO^56^ to select those nonzero groups. The group LASSO estimators of dependent parameters, denoted as ${\dot{\mathbf \beta }}_j$ **=** (**β**_*j|*1_, …, **β**_*j*|(*j*–1)_, **β**_*j*|(*j* + 1)_, …, ${\dot{\mathbf \beta }}_{j|d_i}$), where *d*_*j*_ (≪*m*) is the number of the most significant genes that interact with gene *j*, can be obtained by minimizing the following penalized weighted least-square criterion,

3 $$L_{1}\left( {{\dot{\mathbf \beta }}_{j},\lambda _{j}} \right) = \left( {{\bf{Y}}_{j} - a_{j} - X_{j}^{T}{\mathbf{b}}_{j}} \right)^{T}{\mathbf{Z}}_{j}\left( {{\bf{Y}}_{j} - a_{j} - X_{j}^{T}{\mathbf{b}}_{j}} \right) + \lambda _{1j}\mathop {\sum }\limits_{j\prime = 1,j\prime \ne j}^{m} \left\| {\beta _{j|j\prime }} \right\|_{2},$$

where **y**_*j*_ *=* (*y*_*j*_(*E*_1_), …, *y*_*j*_(*E*_*N*_)), **y**_*j*_ *=* (*y*_*j*_(*E*_1_), …, *y*_*j*_(*E*_*N*_)), **a**_*j*_ *=* (*a*_*j*_(*E*_1_), …, *a*_*j*_(*E*_*N*_)), and **b**_*j*_ *=* (**b**_*j*_(*E*_1_), …, **b**_*j*_(*E*_*N*_)); *λ*_1*i*_ is a penalty parameter determined by BIC or extended BIC; and **Z**_*j*_ = diag{*z*_*j*_(*E*_1_), …, *z*_*j*_(*E*_*N*_)} where *z*_*j*_(*E*_*i*_) is a prescribed nonnegative weight function on [*E*_1_, *E*_*N*_] with boundary conditions *z*_*j*_(*E*_1_) = *z*_*j*_(*E*_*N*_) = 0. This weight function is used to speed up the rate of convergence.

### Optimizing the topological structure of gene co-expression networks

Through variable selection, we detect the most significant incoming links (*d*_*j*_ ≪ *m*) for each gene *j* that constitutes the qdODEs of Eq. (1). By replacing *m* by *d*_*j*_, these ODEs are modified as

4 $$\frac{{dM_{ji}}}{{dE_i}} = g_j\left( {M_{ji}\left( {E_i} \right):{\mathrm{\Theta }}_j} \right) + \mathop {\sum }\limits_{j^\prime = 1,j^\prime \ne j}^{d_j} g_{j|j^\prime }\left( {M_{j^\prime i}\left( {E_i} \right):{\mathrm{\Theta }}_{j|j^\prime }} \right),\,j = 1, \ldots ,m;i = 1, \ldots ,N,$$

which are a sparse version that represents the full model of incoming links for each gene, but with no constraint on the number of outgoing links and, therefore, the dimension of the network. We formulate a likelihood approach to estimate the modified ODEs. Let *ϕ* = (**μ**; **Σ**) ∈ **Φ** denote all model parameters. The likelihood function of **ϕ** given these data is written as

5 $${\cal{L}}\left( {\mathbf{\mu}};{\mathbf{\Sigma}} \right) = f\left( {\boldsymbol{y}_{1}}, \ldots ,{\boldsymbol{y}_{m}}|{\mathbf{\mu}_{1}}, \ldots ,{\mathbf{\mu}_{m}};{\mathbf{\Sigma}} \right),$$

where *f*(·) is the *N*-dimensional *m*-variate normal distribution for *m* gene across *N* samples with mean vector **M**,

6 $${\mathbf{\mu}} = \left( {{\mathbf{\mu}}_1; \ldots ;{\mathbf{\mu}}_m} \right) = \left( {\mu_1\left( {E_1} \right), \ldots ,{\mu}_1\left( {E_n} \right); \ldots ;{\mu}_m\left( {E_1} \right), \ldots ,{\mu}_m\left( {E_n} \right)} \right),$$

and covariance matrix **Σ**,

7 $$\Sigma = \left( {\begin{array}{*{20}{c}} {\Sigma _1} & \cdots & {\Sigma _{1m}} \\ \vdots & \ddots & \vdots \\ {\Sigma _{m1}} & \cdots & {\Sigma _m} \end{array}} \right)$$

In Eq. (6), *μ*_*j*_(*E*_*i*_), the mean value of the expression of gene *j* at sample *i*, whose derivative contains *g*_*j*_(·) and *g*_*j|j*′_(·) specified by the modified qdODEs in Eq. (4), is modeled by B-spline function and estimated by standard fourth-order Runge-Kutta algorithms. Since B-spline nonparametric functions are intergrable, we can calculate *G*_*j*_(·) and *G*_*j|j*′_(·). In Eq. (7), **Σ**_*j*_ is the sample-dependent covariance matrix of gene *j*, and ${\mathbf{\Sigma }}_{jj\prime }$ is the sample-dependent covariance matrix between genes *j* and *j*′. We assume that the residual errors of gene expression are independent among samples and that the residual variance of each gene is constant across samples. Thus, **Σ**_*j*_ and ${\mathbf{\Sigma }}_{jj\prime }$ are structured as ${\mathbf{\Sigma }}_j = \sigma _j^2{\mathbf{I}}_n$ and ${\mathbf{\Sigma }}_{jj\prime } = \sigma _{jj\prime }{\mathbf{I}}_n$, respectively, where $\sigma _j^2$ is the residual variance of gene *j* at the same sample, $\sigma _{jj\prime }$ is the residual covariance of genes *j* and *j*′ at the same sample, and **I**_*n*_ is the identity matrix. However, we implement the first-order autoregressive (AR(1)) model to fit the residual covariances of gene expression among different time points at the same individual.^57^

All model parameters **ϕ** can obtain their optimal solution by maximizing the likelihood in Eq. (5), expressed as

8 $${\hat {\mathbf{\phi}}} \in \left\{ {{\mathrm{arg}}\frac{\max}{{\mathbf{\phi}} \in {\mathbf{\Phi }}}{\cal{L}}\left( {{\mathbf{\mu}},\,{\mathbf{\Sigma }}} \right)} \right\}.$$

Intuitively, this maximum likelihood optimization implies an optimal topological structure and organization in which genes interact with each other to maximize the expression level of all genes as a whole. This solution of Eq. (8) establishes the mathematical formulation of Smith and Price’s evolutionary game theory.^24^

### Significance test of gene interactions

One important issue for network reconstruction is how to statistically test the significance of edges as the measure of associations between nodes. We propose a likelihood ratio approach for network test. Under the null hypothesis that all genes are independent from each other, the rate of expression change for each gene can be formulated by a reduced system of ODEs, expressed as

9 $$\frac{{dM_{ji}}}{{dE_i}} = g_j\left( {M_{ji}\left( {E_i} \right):{\mathrm{\Theta }}_j} \right),\,j = 1, \ldots ,m;i = 1, \ldots ,N$$

which is contrasted to the full system of ODEs in Eq. (4) as the alternative hypothesis stating that at least one gene interaction in the network is significant. We calculate the likelihood values under the null and alternative hypotheses and their log-likelihood ratio (LR) as a test statistic. A network-wise critical threshold can be determined by permutation tests. This procedure includes (i) shuffling sample-varying expression data among genes to make a new data, (ii) calculating the LR value based on this new data, (iii) repeating (i) and (ii) many times (say 1000), and (iv) detecting the 95% percentile of these 1000 LR values which is the cutoff for the significance test of networks.

### Environment-perturbed networks

Genetic networks may be activated when the organism experiences environmental change. Suppose that gene co-expression changes from one sample (say *i*_1_) to next (say *i*_2_) due to differences in the internal environment of samples. The amount of this change can be estimated by integrating the dependent expression component of qdODEs in Eq. (4) from $E_{i_1}$ to $E_{i_2}$, expressed as

10 $${\mathrm{\Delta }}_{j|j^\prime 12} = \mathop {\int }\limits_{E_{i_1}}^{E_{i_2}} g_{j|j^\prime }\left( {M_{j^\prime i}\left( {E_i} \right):{\mathrm{\Theta }}_{j|j^\prime }} \right)dE_t,$$

which quantifies the expression difference of gene *j* regulated by gene *j*′ by assuming that sample transport virtually from *i*_1_ to *i*_2_. GRNs reconstructed from ${\mathrm{\Delta }}_{j|j^\prime 12}$ (*j* ≠ *j*′ = 1, …, *m*) reflect the alterations of gene co-expression in response to environmental change, which are called environment-perturbed GRNs. Based on this definition, we can reconstruct treatment-, outcome-, development, or signal-perturbed networks to better understand the genomic mechanisms underlying cellular, physiological, and ecological processes.

### Reporting summary

Further information on research design is available in the Nature Research Reporting Summary linked to this article.

## Supplementary information

Supplementary Figures

Supplementary Table

reporting Summary

## Data Availability

The data and code that support the findings of this study are available from https://github.com/chencxxy or can be requested from the corresponding author.
