# Supplementary material for: An omnidirectional visualization model of personalized gene regulatory networks
Source: NPJ Syst Biol Appl. 2019 Oct 11;5:38. doi: 10.1038/s41540-019-0116-1 (PMC6789114; doi:10.1038/s41540-019-0116-1)
Supplement: Supplementary file 1 — Supplementary Figures [file 41540_2019_116_MOESM1_ESM.docx]

**Supplementary Figure Legends**


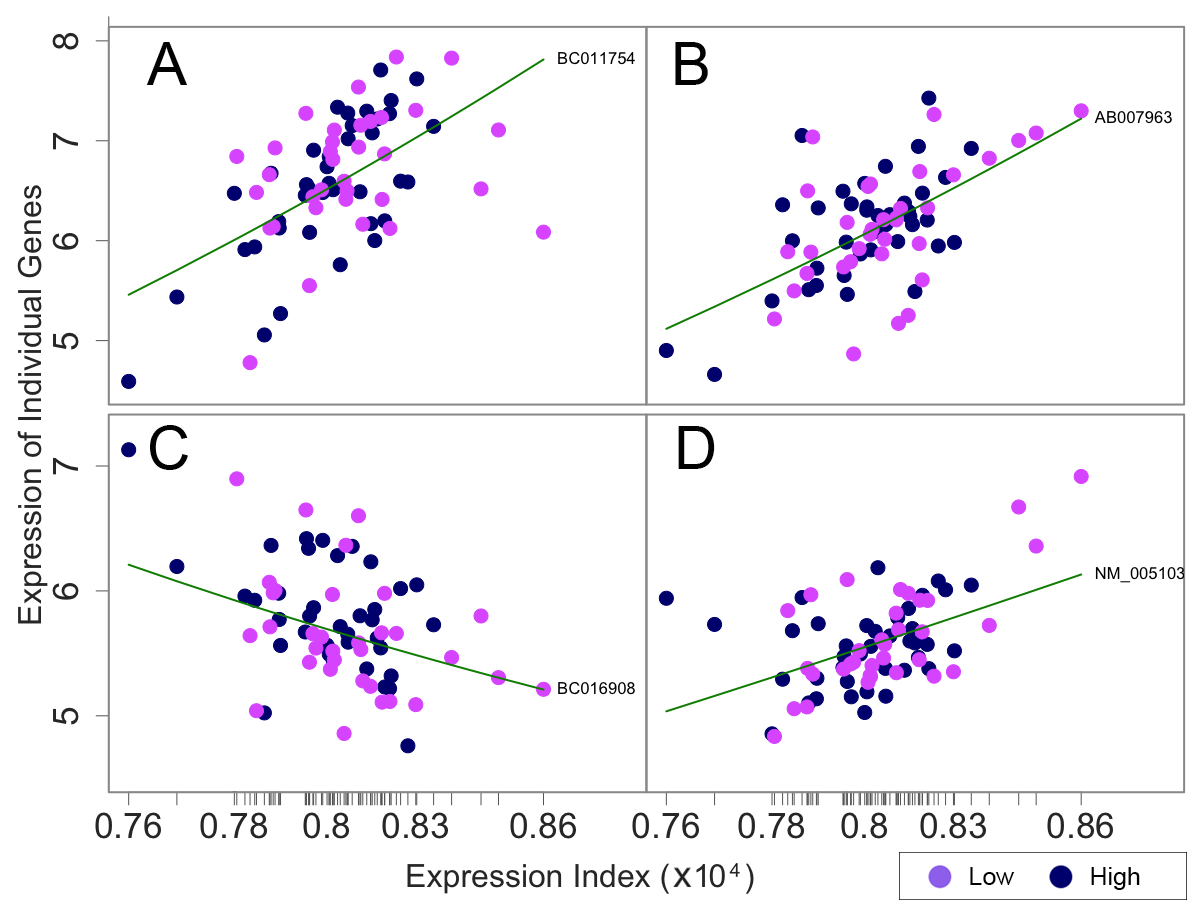


**Figure S1.** The fitness of a power equation as a function of expression index (EI) (green line) to the observed expression levels of four genes, BC011754 (**A**), AB007963 (**B**), BC016908 (**C**), and NM_005103 (**D**) across 73 rabbit samples. Samples include three to six rabbits under each of two blood flows, low (purple circles) and high (dark circles), measured at each of eight time points (hour 2 and days 1, 3, 7, 14, 30, 90, and 180) post-operation. Ticks on the x-axis represent the positions of each sample in terms of its EI.

**
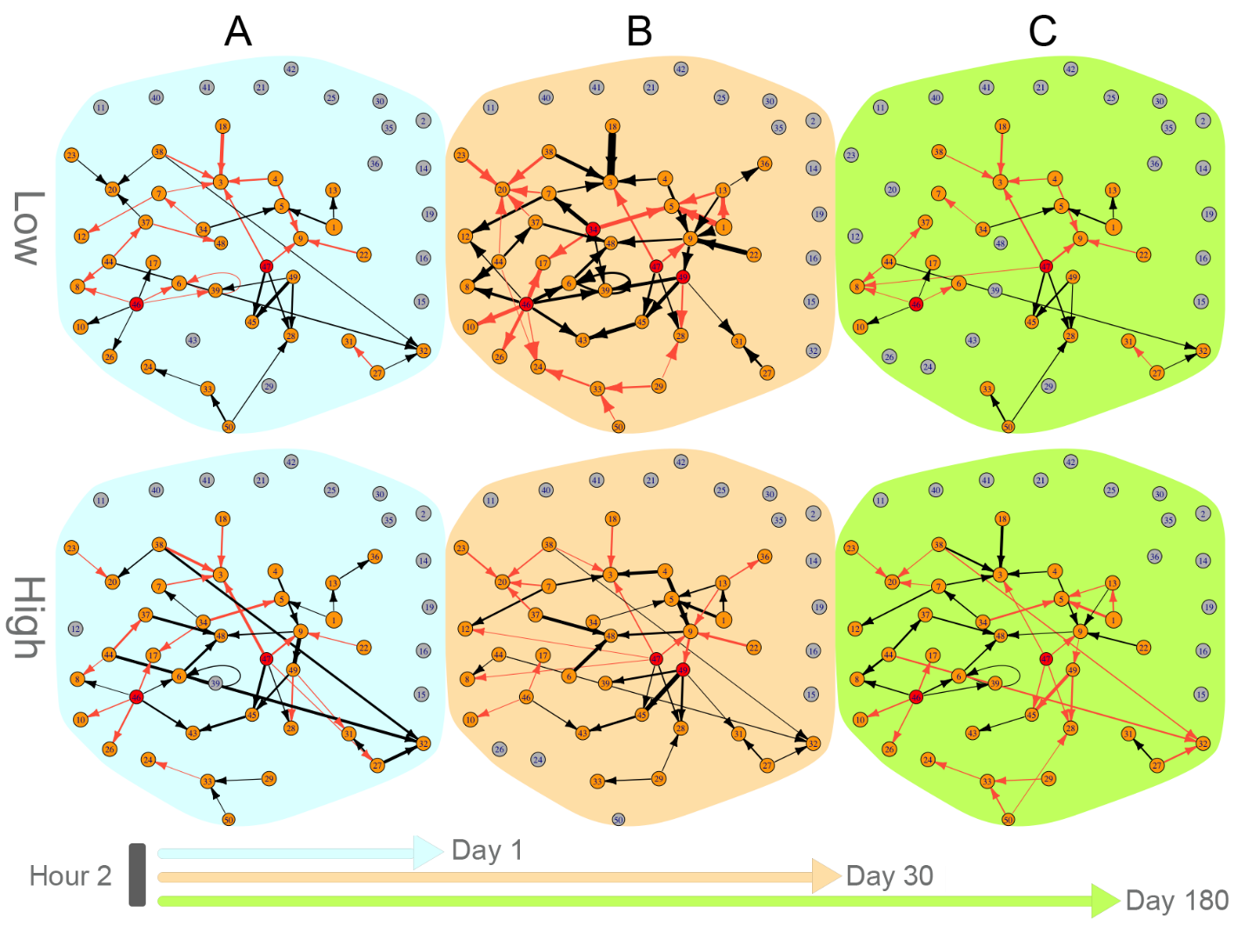
**

**Figure S2.** Development-perturbed networks at the module level under low flow (upper panel) and high flow (lower panel) of rabbit vein grafting experiment in response to developmental stimuli from hour 2 to day 1 (**A**), day 30 (**B**), and 180 (**C**) post-operation. Numbers in small circles (each denoted as a node of the graph) represent module IDs. Red and black arrows denote the direction by a gene promotes and inhibits other genes, respectively, and the thickness of an arrowed line is proportional to the strength of promotion or inhibition. A proportion of modules are unlinked, suggesting that they are neutral to each other and other linked genes. Dark red circles denote hub modules with higher connectivity than the average number of links among all modules.

**
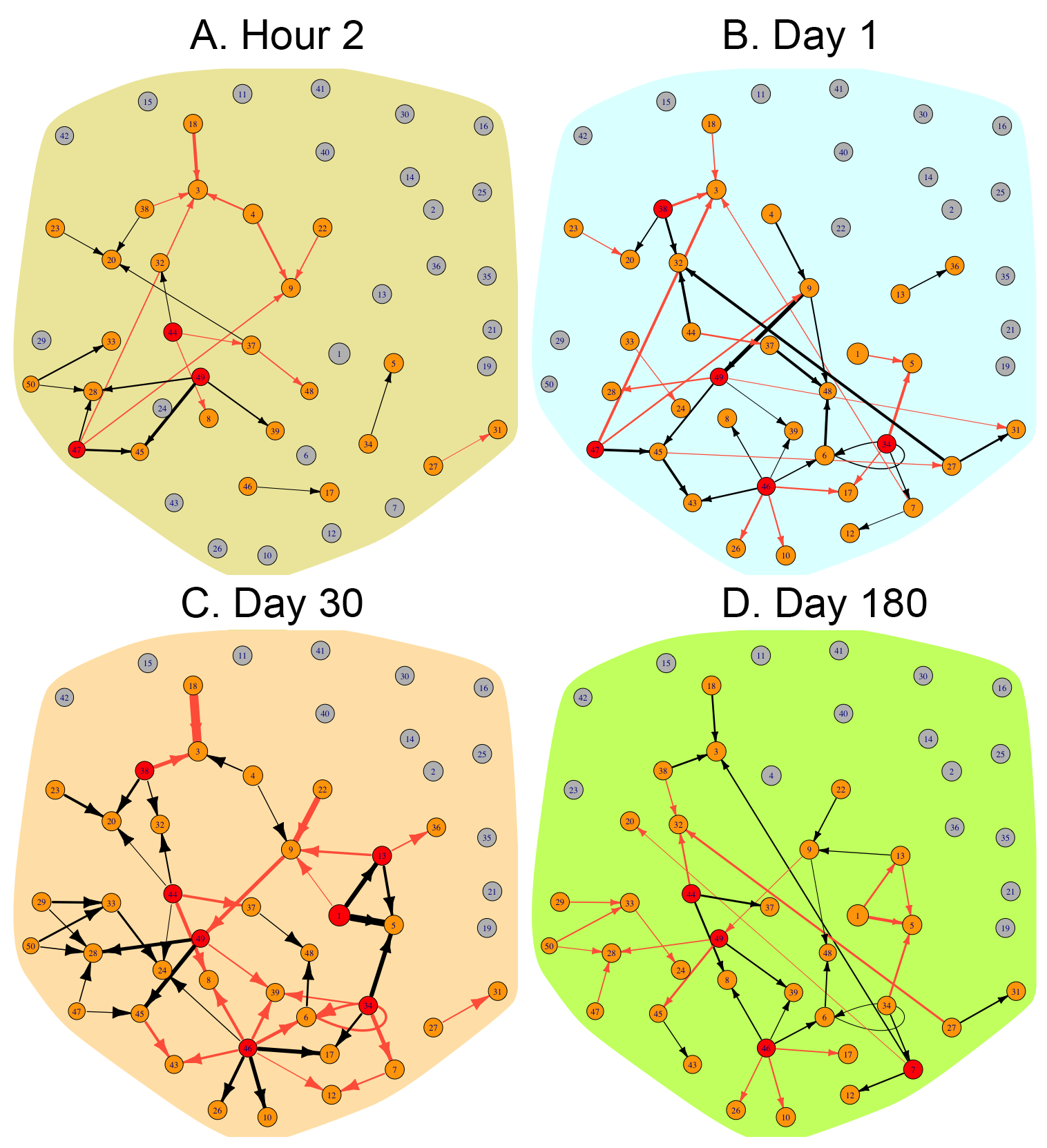
**

**Figure S3.** Flow-perturbed networks at the module level from slow to high flows of grafted rabbits at hour 2 (**A**), day 1 (**B**), day 30 (**C**), and day 180 (**D**) post-operation. Numbers in small circles (each denoted as a node of the graph) represent module IDs. Red and black arrows denote the direction by a gene promotes and inhibits other genes, respectively, and the thickness of an arrowed line is proportional to the strength of promotion or inhibition. A proportion of modules are unlinked, suggesting that they are neutral to each other and other linked genes. Dark red circles denote hub modules with higher connectivity than the average number of links among all modules.

**
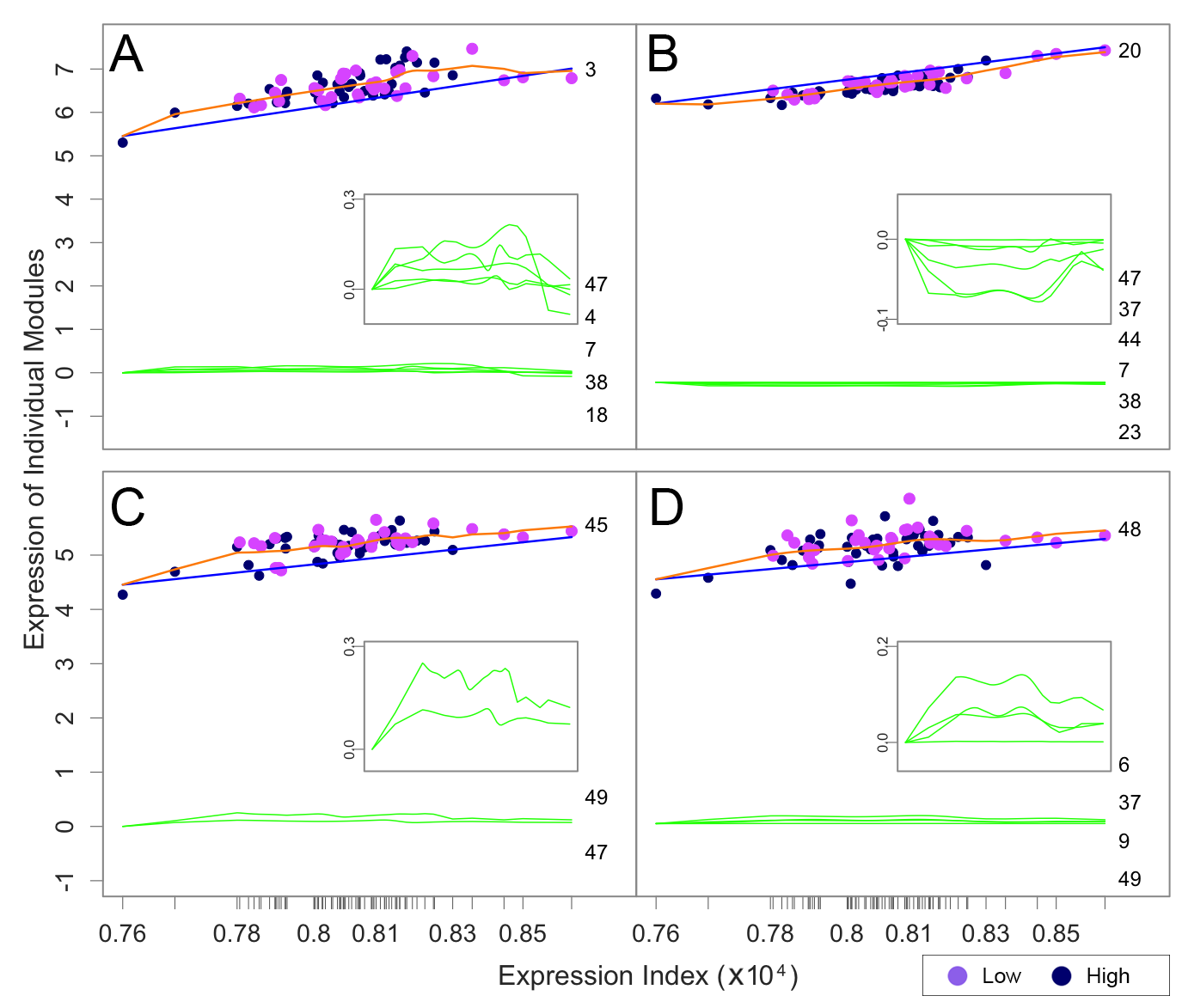
**

**Figure S4.** Overall fitted curves of gene expression (orange line) from modules 3 (**A**), 20 (**B**), 45 (**C**), and 48 (**D**) by a system of qdODEs as a function of expression index (EI) in the rabbit vein grafting experiment. Each dot denotes a sample representing a rabbit under a blood flow, low (purple) or high (dark), measured at a time point (hour 2 and days 1, 3, 7, 14, 30, 90, and 180) post-operation. The overall expression curve of each module is decomposed into its endogenous expression curve (blue line) and exogenous expression curves (green lines) exerted by a set of other modules (listed by IDs). Exogenous expression curves are better displayed by a small plot within each large plot. Value 0 at y-axis is a cut-off point that describes how a focal module is regulated by other modules: Greater than 0 for promotion, less than 0 for inhibition, and zero for neutrality. Ticks on the x-axis represent the positions of each sample in terms of its EI.
